# ITrace study on the Effects of Phacoemulsification with 90° Transparent Corneal Incision on Corneal Astigmatism and Visual Acuity in Patients Undergoing Cataract Surgery

**DOI:** 10.12669/pjms.40.11.9029

**Published:** 2024-12

**Authors:** Yuanyuan Tian, Shuang Jiang, Huafeng Zhang, Jia Zhang

**Affiliations:** 1 Yuanyuan Tian Department of Ophthalmology, Depeartment of Comprehensive Eye Diseas, Third Affiliated Hospital of Jinzhou Medical University, Jinzhou, 121000, Liaoning, China; 2 Shuang Jiang Department of Ophthalmology, Third Affiliated Hospital of Jinzhou Medical University, Jinzhou, 121000, Liaoning, China; 3 Huafeng Zhang Depeartment of Comprehensive Eye Diseas, Gaocheng Aier Shijia Eye Hospital, Gaocheng, Hebei, 052160, China; 4 Jia Zhang Depeartment of Comprehensive Eye Diseas, Gaocheng Aier Shijia Eye Hospital, Gaocheng, Hebei, 052160, China

**Keywords:** Transparent corneal incision, Phacoemulsification, Cataract surgery, Corneal astigmatism, Visual acuity

## Abstract

**Objective:**

To investigate the effects of phacoemulsification with 90° transparent corneal incision on corneal astigmatism and visual acuity in patients undergoing cataract surgery.

**Methods:**

This was a retrospective study. Sixty patients with age-related cataract and corneal astigmatism value ≤ 0.75D admitted to Gaocheng Aier Shijia Eye Hospital from June 2023 to October 2023 were included and divided into the observation group(with-the-rule astigmatism) and the control group(against-the-rule astigmatism), with 30 eyes each. Both groups were given a 90° transparent corneal incision, and the general data of the two groups were compared before surgery, one week after surgery, one month after surgery, and three months after surgery.

**Results:**

Before surgery, UCVA and BCVA were compared between the two groups (*p>* 0.05); no statistically significant difference was observed in corneal astigmatism between the two groups (*p>* 0.05); no statistically significant difference was observed in the axial direction of corneal astigmatism between the two groups before surgery(p> 0.05). Inter-group comparison: UCVA and BCVA in the two groups were significantly increased one week and one month after surgery, with those in the observation group being higher than those in the control group(*p<* 0.05); the corneal astigmatism in the observation group was lower than that in the control group at one week and one month after surgery (*p<* 0.05).

**Conclusion:**

A 90° transparent corneal incision may result in various benefits during phacoemulsification, such as correcting corneal astigmatism in patients with compliant cataracts, promoting early postoperative visual recovery, and stabilizing axial position after surgery.

## INTRODUCTION

Cataract, as a common eye disease, tends to make inroads on middle-aged and elderly people and has a high blindness rate, which has had a serious impact on patients’ daily life and work.^1^,^2^ Surgical methods such as phacoemulsification can effectively alleviate the condition of patients and improve their astigmatism^3^ Studies have shown^4^,^5^ that there is a certain relationship between the method and location of surgical incisions and corneal astigmatism in cataract patients. Some scholars have also concluded that^6^ the size of corneal morphological changes and the severity of astigmatism after cataract surgery are affected by factors such as preoperative astigmatism, axial direction, incision size, and incision configuration. In the treatment of cataract, the focus has gradually changed to refractive surgery to correct preoperative corneal astigmatism, reduce postoperative corneal astigmatism, and improve postoperative visual quality.^7^

However, most of the current clinical studies focus on the effects of different coaxial incisions on visual acuity recovery of patients. Few studies have been conducted on the effects of the same incision location on astigmatism in different coaxial positions(with-the-rule astigmatism and against-the-rule astigmatism). To this end, we investigated the effects of phacoemulsification with 90° transparent corneal incision on corneal astigmatism and visual acuity in patients undergoing cataract surgery.

## METHODS

This was a retrospective study. Sixty patients with age-related cataract and corneal astigmatism value ≤ 0.75D admitted to Gaocheng Aier Shijia Eye Hospital from June 2023 to October 2023 were included and divided into the observation group(with-the-rule astigmatism) and the control group(against-the-rule astigmatism), with 30 eyes each according to the same treatment methods. No statistically significant difference was observed in the general baseline data between the two groups(*p>* 0.05) (Table-I).

**Table-I:** Comparison of general data between the two groups.

Group	Number of cases (n)	Gender (Male/Female)	Average age (years old)	Energy-little classification		

				Class-II	Class-III	Class-IV
Observation group	30	19/11	71.13±4.24	8	17	5
Control group	30	17/13	70.83±4.36	9	18	3
*χ*^2^/*t*		0.278	0.270	0.587		
*p*		0.598	0.788	0.746		

### Ethical Approval:

The study was approved by the Institutional Ethics Committee of Gaocheng Aier Shijia Eye Hospital (No.:EYEGCYY-20200105-01; Date: January 5, 2020), and written informed consent was obtained from all participants.

### Inclusion criteria:

- Patients who met the diagnosis and treatment standards for cataract surgery.^8^
- Aged 60~80 years old.
- Those were diagnosed with age-related cataracts and corneal astigmatism value ≤ 0.75D detected by iTrace.
- Those who were willing to participate in this study and signed the informed consent form.

### Exclusion criteria:

- Patients with other anterior segment and posterior segment diseases.
- Those with diabetes and hypertension.
- Those with keratitis, glaucoma and other ophthalmic diseases.
- Those with severe intraoperative or postoperative complications.
- Those with a history of previous surgery.

Both group made a 90° transparent corneal incision according to the principal meridian of maximum refractive power, respectively. The operation was as follows: The patient was placed in a supine position with routine ophthalmic disinfection of the surgical eye, a sterile towel was laid, and the eyelid was opened with an eyelid opener, 0.4% procaine hydrochloride eye drops were used for topical anesthesia, and povidone iodine was used to wash the conjunctival sac. An auxiliary incision of 1 mm in the limbus was made under the microscope at 9 o’clock position with a 15° emulsification knife, and sodium hyaluronate viscoelastic was injected in the anterior chamber.

At 12 o’clock position, a 3.0 mm incision was made in the corneosclera with a 3.0mm emulsifying knife. Continuous annular capsulorhexis (about 5.5 mm×5.5 mm) was performed, water separation, and phacoemulsification was performed to remove the crystal nucleus and cortex. The posterior capsule was polished and sodium hyaluronate was injected into the anterior chamber and lens pouch. Intraocular lens was implanted into the pouch, sodium hyaluronate was sucked out of the anterior chamber and the lens pouch, and the incision was watertight. No leakage was detected in the incision, the anterior chamber was of medium depth, and the IOP was Tn. Tobramycin and dexamethasone eye ointment was applied to the conjunctival sac, and the operative eye was bandaged with sterile gauze. All the operations were performed by the same group of doctors.

### Observation indicators:

The uncorrected visual acuity (UCVA) and the best corrected visual acuity (BCVA) of the two groups were recorded before surgery, one week after surgery, one month after surgery and three months after surgery.^9^ The corneal astigmatism values of the two groups were measured by iTrace. The axial position of postoperative astigmatism was compared between the two groups, including with-the-rule astigmatism (WTRA) and against-the-rule astigmatism (ATRA).^10^ Mediworks electric focusing digital slit lamp microscope system S390H was used to measure the depth of peripheral anterior chamber and central anterior chamber of the two groups, that is, the distance from the inner surface of cornea to the outer surface of iris and pupil. The two groups of patients were followed up for six months.

### Statistical analysis:

All data in this study were statistically analyzed using SPSS 26.0 software, and measurement data are expressed in (*χ̅*±*S*). The confidence interval was 95%, intragroup comparisons were made by the paired t-test and intergroup comparisons by the independent-sample t-test. Comparisons between groups were performed using analysis of variance, count data were expressed as [cases (%)], and χ^2^ test was used for rate comparison. P< 0.05 was considered a statistically significant difference.

## RESULTS

The general clinical data of the two groups of patients were compared (*p>* 0.05). Table-I Before surgery, UCVA and BCVA were compared between the two groups (*p>* 0.05). *Intra-group comparison:* UCVA and BCVA in the two groups were increased one week, one month, and three months after surgery compared to those before surgery; One week after surgery, UCVA and BCVA were significantly higher than before surgery (*p<* 0.05), and UCVA in the observation group one month after surgery were higher than those before surgery and one week after surgery (*p<* 0.05); One week after surgery, BCVA in both groups was improved compared with before surgery, and that in the observation group was significantly higher than that in the control group (*p<* 0.05); *Inter-group comparison:* UCVA and BCVA in the two groups were significantly increased one week and one month after surgery, with those in the observation group being higher than those in the control group (*p<* 0.05). Table-II

**Table-II:** Comparison of UCVA and BCVA between the two groups (*χ̅*±*S*, LogMAR).

		UCVA				BCVA			

		Before surgery	one week after surgery	one month after surgery	three months after surgery	Before surgery	one week after surgery	one month after surgery	three months after surgery
Observation group	30	1.15±0.43^*^	0.16±0.16^a^	0.07±0.10^ab^	0.05±0.07^ab^	0.71±0.28	0.00±0.02^a^	0.00±0.01^a^	0.00±0.01^a^
Control group	30	1.17±0.40	0.29±0.17^a^	0.18±0.11^ab^	0.09±0.08^abc^	0.80±0.37	0.04±0.10^a^	0.03±0.07^a^	0.00±0.02^ab^
t		0.186	3.126	3.768	2.184	1.061	2.050	2.062	0.705
p		0.853	0.003	0.000	0.033	0.293	0.045	0.044	0.484

***Note:***

a p< 0.05 compared with before surgery, ^b^p< 0.05 compared with one week after surgery.

Before surgery, no statistically significant difference was observed in corneal astigmatism between the two groups (*p>* 0.05). A pairwise comparison was further performed. Intra-group comparison: The corneal astigmatism of the two groups was lower than that before surgery at one week and one month after surgery, and that at one month after surgery was significantly lower than that at one week after surgery (*p<* 0.05). Inter-group comparison: The corneal astigmatism in the observation group was lower than that in the control group at one week and one month after surgery (*p<* 0.05). Table-III

**Table-III:** Comparison of corneal astigmatism between the two groups at different time points (*χ̅*±*S*, °C).

Group	Number of cases	one week after surgery	one month after surgery	three months after surgery
Observation group	30	2.41±0.50^a^	1.21±0.47^ab^	1.10±0.24^b^
Control group	30	2.87±0.55^a^	1.98±0.52^ab^	1.21±0.28^abc^
*t*		3.383	6.032	1.613
*p*		0.001	0.000	0.112

***Note:***

a p< 0.05 compared with before surgery,

b p< 0.05 compared with one week after surgery, ^c^ p< 0.05 compared with one month after surgery.

Before surgery, no statistically significant difference was observed in the axial direction of corneal astigmatism between the two groups before surgery (*p>* 0.05). A pairwise comparison was further performed. Intra-group comparison: No statistically significant difference was observed in the axial direction of corneal astigmatism in the observation group one week, one month and three months after surgery compared with that before surgery (*p>* 0.05); The axial changes in the control group were significant (*p>* 0.05). Inter-group comparison: The axial changes of corneal astigmatism in the observation group were less than those in the control group at each time point after surgery (*p<* 0.05). Table-IV

**Table-IV:** Comparison of axial changes in corneal astigmatism between the two groups at different time points (n).

Group	Number of cases	Before surgery	One week after surgery	One month after surgery	Three months after surgery	χ^2^	p

		WTRA/ATRA	WTRA/ATRA	WTRA/ATRA	WTRA/ATRA		
Observation group	30	11/19	14/16	13/17	10/20	1.212	0.750
Control group	30	11/19	19/11^a^	21/9 ^a^	19/11^a^	8.091	0.044

***Note:***

a p< 0.05 compared with before surgery.

Before surgery, no statistically significant difference was observed in the anterior chamber depth between the two groups (*p>* 0.05). A pairwise comparison was further performed. Intra-group comparison: The anterior chamber depth in both groups was significantly increased at one month and three months after surgery (*p<* 0.05), and that at three months after surgery was significantly higher than that at one month after surgery (all *p<* 0.05). Inter-group comparison: The anterior chamber depth in the two groups increased at one week, one month and three months after surgery, but with no statistically significant difference between the two groups (*p>* 0.05). Table-V

**Table-V:** Comparison of changes in anterior chamber depth between the two groups at different time points (*χ̅*±*S*, mm).

Group	Number of cases	Central anterior chamber depth				Peripheral anterior chamber depth			

		Before surgery	one week after surgery	one month after surgery	three months after surgery	Before surgery	one week after surgery	one month after surgery	three months after surgery
Observation group	30	2.74±0.47	3.30±0.52	3.62±0.38	3.94±0.20	1.51±0.33	1.85±0.28	2.11±0.22	2.24±0.20
Control group	30	2.68±0.39	3.26±0.46	3.59±0.34	3.80±0.35	1.48±0.32	1.80±0.33	2.05±0.27	2.19±0.24
*t*		0.537	0.316	0.325	1.910	0.360	0.635	0.995	0.894
*p*		0.593	0.753	0.747	0.061	0.720	0.528	0.324	0.375

## DISCUSSION

It was found that the UCVA and BCVA of both groups were improved one week, one month, and three months after surgery compared with those before surgery. The UCVA of both groups increased at one month and one week after surgery(*p<* 0.05), while that at three months after surgery decreased compared with that at one week and one month after surgery (*p>* 0.05). It shows that the UCVA and BCVA of the two groups are improved one week and one month after surgery, and have little effect on the visual acuity three months after surgery. And that cataract patients with 90° transparent corneal incision and astigmatism can get better postoperative visual acuity, it is more beneficial for patients to recover early after incision.^11^

This is highly similar to the results obtained by Gupta S et al.^12^ that small incisions are beneficial to the short-term recovery of vision in cataract patients with WTRA. It was found that the corneal astigmatism of the two groups decreased one week and one month after surgery (*p<* 0.05), and there was no statistical difference between the two groups in three months after surgery (*p>* 0.05). It is suggested that the early postoperative corneal astigmatism of cataract patients with WTRA before surgery can recover faster than that of those with ATRA.

Cataract is one of the common blinding diseases in the world, and cataract patients often have varying degrees of astigmatism after surgery. In the absence of effective treatment measures, it will have adverse effects on patients’ visual function.^13^,^14^ It is found that^15^ a 90° incision produces less astigmatism, showing with-the-rule astigmatism(WTRA), and choose 90° transparent corneal incision have better relaxation effect because the curvature of the meridian cornea where the incision is located tends to be flat. In this way, they can effectively reduce the refractive power of the meridian and reduce the degree of corneal astigmatism. However, in patients with ATRA, the early recovery of corneal astigmatism after surgery is slow because of its large vertical refractive power, but the long-term impact is small. This is consistent with the conclusion of Li PP et al.^16^,^17^ that choosing a steep corneal incision is beneficial to alleviate the early corneal astigmatism of cataract patients with WTRA before surgery.

It was found in this study that the corneal thickness around the incision of the two groups decreased one week after surgery, and that in the observation group was significantly lower than that in the control group (*p<* 0.05). There was no significant difference between the two groups in one month and three months after surgery (*p>* 0.05), indicating that the corneal thickness around the incision of the two groups decreased within one week after surgery, and the decreasing effect of the patients with regular astigmatism before surgery was more obvious, but there was no difference in long-term effect.

This is because corneal edema and tissue inflammation will be caused by incision injury in the early postoperative period. With the passage of time, the corneal thickness around the incision of the patient will gradually return to the preoperative level. Méndez EA et al.^9^ found that phacoemulsification with transparent corneal incision for cataract patients with different astigmatism axes has a certain effect on corneal diopter and corneal astigmatism in the short term, while patients with WTRA recover faster in the short term. It was also found that the corneal astigmatism in the control group was axial compared with that in the observation group (*p>* 0.05).

It was also found that the axial direction of corneal astigmatism of the control group was significantly changed at each time point before and after surgery (*p>* 0.05), and the axial changes of corneal astigmatism in the observation group were smaller than those in the control group at various time points before and after surgery (*p<* 0.05), indicating that the axial deviations of corneal astigmatism in patients with WTRA before and after surgery were small and stable. Many studies have found that^18^ the postoperative astigmatism axis of cataract patients is mainly ATRA, which is mainly caused by the aging of cataract patients and the relaxation of eyelid skin.

The preoperative axial alignment is consistent with the postoperative ATRA axis, so the incision axis is relatively stable, which is consistent with the conclusions of Wang JD et al.^19^ because cataract surgery is to replace intraocular lens, it is inevitable to deepen the anterior chamber. However, the change of anterior chamber depth is easily affected by corneal thickness. In this study, the anterior chamber depth of patients in both groups increased at each time point after surgery, but there was no statistically significant difference between the two groups (*p>* 0.05), indicating that the anterior chamber depth of patients would increase at the initial stage after surgery due to corneal thickening, but different astigmatism axes had little effect on the anterior depth. It is highly consistent with the conclusion of Han T et al.^20^ that the axial astigmatism has little influence on the depth in front of the patient.

### Limitations:

It includes small sample size, small course of treatment, and short follow-up time. It still needs further clinical research to observe the long-term clinical effect of Phacoemulsification with 90° Transparent Corneal Incision in patients undergoing cataract surgery, so as to apply a better scheme to patients in need.

## CONCLUSIONS

A 90° transparent corneal incision may result in various benefits during phacoemulsification, such as correcting corneal astigmatism in patients with compliant cataracts, promoting early postoperative visual recovery, and stabilizing axial position after surgery. It has a positive effect on improving patients’ early visual acuity and promoting rapid rehabilitation.

### Authors’ Contributions:

**YT** and **SJ:** Carried out the studies, data and drafted the manuscript and are responsible and

**HZ** and **JZ:** Performed the statistical analysis and participated in its design. Critical Review

All authors read, approved the final manuscript and are accountable for the accuracy integrity of the work.
